# United States Veterinary Medical Association

**Published:** 1897-10

**Authors:** 


### UNITED STATES VETERINARY MEDICAL ASSOCIATION.

### List of Officers and Committee Appointments for 1897–98.

List of Officers. D. E. Salmon, President; T. B. Rayner, W. C. Royen, and A. T. Peters, Vice-Presidents; S. Stewart, Secretary; William Herbert Lowe, Treasurer.

List of Committees. *Executive.* T. S. Butler (Chairman), F. H. Osgood, W. Horace Hoskins, W. L. Williams, T. J. Turner, M. Stalker, M. H. Reynolds.

*Publication.* W. L. Williams (Chairman), N. P. Hinkley, R. R. Bell, W. B. Niles, S. Stewart.

*Finance.* M. Stalker (Chairman), M. H. McKillip, S. Stewart.

*Intelligence and Education.* Leonard Pearson (Chairman), W. B. Niles, John M. Parker, Olof Schwartzkopf, R. R. Dinwiddie.

*Diseases.* A. T. Peters (Chairman), E. P. Niles, W. H. Dalrymple, Joseph Hughes, J. F. Winchester.

*Resolutions.* A. W. Clement (Chairman), Leonard Pearson, C. A. Cary. A. T. Peters, S. Stewart.

